# Pre- and Postsynaptic Role of Dopamine D2 Receptor DD2R in *Drosophila* Olfactory Associative Learning

**DOI:** 10.3390/biology3040831

**Published:** 2014-11-21

**Authors:** Cheng Qi, Daewoo Lee

**Affiliations:** Neuroscience Program, Department of Biological Sciences, Ohio University, Athens, OH 45701, USA; E-Mail: chengqi010@gmail.com

**Keywords:** *Drosophila* larvae, aversive learning, appetitive learning, dopaminergic neurons, mushroom body

## Abstract

Dopaminergic neurons in *Drosophila* play critical roles in diverse brain functions such as motor control, arousal, learning, and memory. Using genetic and behavioral approaches, it has been firmly established that proper dopamine signaling is required for olfactory classical conditioning (e.g., aversive and appetitive learning). Dopamine mediates its functions through interaction with its receptors. There are two different types of dopamine receptors in *Drosophila*: D1-like (dDA1, DAMB) and D2-like receptors (DD2R). Currently, no study has attempted to characterize the role of DD2R in *Drosophila* learning and memory. Using a DD2R-RNAi transgenic line, we have examined the role of DD2R, expressed in dopamine neurons (*i.e.*, the presynaptic DD2R autoreceptor), in larval olfactory learning. The function of postsynaptic DD2R expressed in mushroom body (MB) was also studied as MB is the center for *Drosophila* learning, with a function analogous to that of the mammalian hippocampus. Our results showed that suppression of presynaptic DD2R autoreceptors impairs both appetitive and aversive learning. Similarly, postsynaptic DD2R in MB neurons appears to be involved in both appetitive and aversive learning. The data confirm, for the first time, that DD2R plays an important role in *Drosophila* olfactory learning.

## 1. Introduction

Dopamine (DA) is an important neurotransmitter mediating a variety of brain functions including locomotion, reward, awareness, learning and memory, and cognition [1,2,3]. Genetic and pharmacological studies revealed that the dopaminergic system in the fruit fly *Drosophila melanogaster* plays multiple roles in motor function and associative learning [3,4]. Using the sophisticated genetic tools available for the fruit fly, it has been firmly established that release of dopamine is required for associative learning in *Drosophila* adults and larvae [5,6,7,8]. Dopaminergic neural circuits mediating olfactory learning have been also characterized in the fruit fly brain [9,10].

DA mediates its physiological functions through interaction with its receptors. Analysis of the primary structure of the DA receptors revealed that those receptors belong to the G-protein coupled receptor (GPCR) family [11,12]. Generally, DA receptors can be divided into two families in vertebrates [2]. The D1-like receptor family stimulates cAMP production by activation of the receptor-coupled G_s_ subunit of G proteins. The D2-like receptor family belongs to the pertussis toxin (PTX)-sensitive G protein (*i.e.*, G_i_ and G_o_)-coupled receptor (GPCR) superfamily. Therefore, actions of D2-like receptor members have been characterized as inhibitory. In relation to regulation of DA signaling, Aghajanian and Bunney [13] found a very interesting feature: DA neurons possess receptors for their own transmitter, dopamine, at their synaptic nerve terminals. These DA autoreceptors (autoR) function as self-inhibitory regulators [14,15].

In *Drosophila*, there are four DA receptors—dDA1, DAMB, DopEcR, and DD2R—that have been cloned and characterized. Two DA receptors (dDA1, DAMB) were cloned first [16,17,18] and appear to be members of the D1-like receptor family on the basis of their ability to stimulate adenylyl cyclase (AC) in a heterologous expression system. In contrast, only one *Drosophila* DA receptor DD2R gene has been identified [19]. Functional expression of the DD2R gene in HEK293 cells indicated that DA caused a marked decrease in forskolin-induced cAMP level, indicating that DD2R belongs to the inhibitory D2-like receptor family. Interestingly, two recent studies [20,21] confirmed the existence of DA autoreceptors in *Drosophila*.

Olfactory associative learning in adult flies requires expression of *Drosophila* D1 receptor dDA1 in the mushroom body, the anatomical center for learning and memory [22]. The dDA1 mutant dumb showed impaired appetitive learning as well as aversive learning. These impaired learning behaviors were fully rescued by expression of the wild-type dDA1 transgene in MB neurons in mutant flies, further confirming the role of *Drosophila* D1-like receptors in learning. However, no previous study has attempted to characterize the role of D2-like DD2R in *Drosophila* learning and memory. Interestingly, there was one study showing that a D2 agonist eticlopride did not disrupt visual learning (e.g., T maze assay) in adult flies [23].

In this study, we chose *Drosophila* larvae carrying DD2R-RNAi transgene to examine the role of D2-like receptors in associative learning. Two different types of tissue-specific drivers were used to examine both presynaptic D2 autoreceptors and postsynaptic D2 receptors. Dopaminergic-specific driver TH-Gal4 was used to induce DD2R-RNAi expression in DA neurons. Since the target of dopaminergic innervation is the mushroom body (MB), the center for learning and memory in *Drosophila*, MB-specific drivers (201Y-Gal4, 30Y-Gal4) were used to down-regulate postsynaptic DD2R combined with DD2R-RNAi transgene. Our results showed that both presynaptic DD2R autoreceptors and postsynaptic receptors are required for aversive and appetitive olfactory learning in *Drosophila* larvae. Potential physiological mechanisms underlying DD2R-mediated learning in *Drosophila* are proposed in the Discussion section.

## 2. Materials and Methods

### 2.1. Fly Strains

Flies were kept in a standard cornmeal/agar medium with 0.4% propionic acid at 25 °C in a 12-h light/dark cycle. The following fly strains were used: wild type (Canton-S), TH-Gal4 (a gift from J. Hirsh, University of Virginia, Charlottesville, VA, USA), UAS-DD2R-RNAi (from Bloomington *Drosophila* Stock Center (BDSC), Bloomington, IN, USA), 201Y-Gal4 (from BDSC), and 30Y-Gal4 (from BDSC). In order to obtain third-instar larvae (92–96 h after egg-laying (AEL)) for learning assays, adult flies were placed in an egg collection bottle with an agar plate carrying yeast paste. Next, flies were allowed to lay eggs for 4 h. Each plate had roughly 100–150 eggs on the surface. The plates were incubated at 21.7 °C. In the late third stage (92–96 h AEL), the larvae were filtered from the food plates using a 500 µm sieve (Standard Test Sieve, Newark Wire Cloth Co., Clifton, NJ, USA) with 15% glucose solution. After rinsing with distilled water, larvae were used for the following tests.

### 2.2. Larval Olfactory Learning

**Figure 1:**
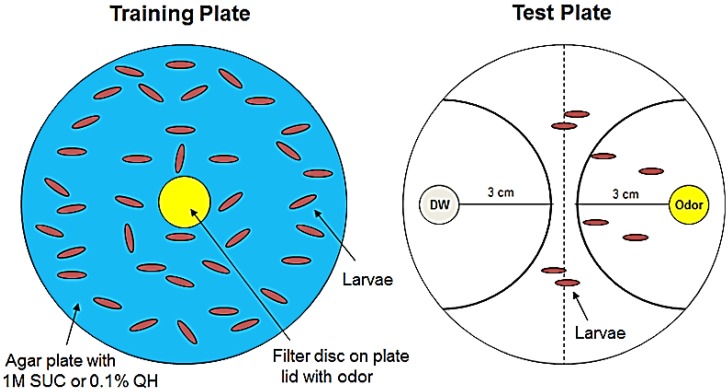
Larval olfactory learning assay. (***Left***) Training: third-instar larvae (92–96 h after egg laying) are trained on a 2.5% agar plate that is covered with 2 mL of either 1 M sucrose solution (SUC, for appetitive learning) or 0.1% quinine hemisulfate solution (QH, for aversive learning). Distilled water (DW) is used as a control. During training time, an odorant (Pentyl Acetate) is placed on a small piece of filter inside the lid. (***Right***) Test: Larvae are rinsed and transferred to the middle line of a new 2.5% agar plate after training. A small piece of filter paper with Pentyl Acetate (PA) is placed on one side of the plate, while the control is the other side. We then counted the number of larvae in the two semicircular areas and calculated the response index (R.I., see text for further detail) after 5 min.

Late third-instar larvae were trained and tested for olfactory learning performance according to the protocols previously described [7,24] (Figure 1). First, the larvae were transferred to the training plate, which is a 100 × 15 mm petri dish (BD Falcon, BD Biosciences, Franklin Lakes, NJ, USA) with 2.5% agar. Training for aversive learning assay, 2 mL 0.1% quinine hemisulfate solution (Sigma-Aldrich Co., St. Louis, MO, USA) was added and spread into a thin layer on the surface of an agar plate; 2 mL 1 M sucrose was used for the appetitive learning assay. Distilled water (DW) was used as a control for both learning assays. Ten microliters of odorant (Pentyl Acetate; Sigma-Aldrich Co., St. Louis, MO, USA) was added on a 1 cm^2^ square filter disk, which was loaded on the inner side of a petri dish lid. Fifty to one hundred larvae were put in one training plate and trained for 30 min.

Larvae were then transferred into the midline of testing plates (Figure 1), which is a 100 × 15 mm petri dish with 2.5% agar. Two plastic lids of 1.5 mL Eppendorf tubes were placed on the opposite sides of the testing plate. The lids served as support for odor-containing filters (1 cm^2^ square). Fifty to one hundred larvae were tested in each round, with odor on one side (2.5 μL) and none on the other side (control). We counted the number of larvae in the two semicircular areas (Figure 1) and calculated the response index (R.I.), 5 min after placing larvae in the test plate. R.I. was calculated with the following equation:

R.I. = [number of larvae in 3 cm semicircle with odor − number of larvae in 3 cm semicircle with DW] / [total number of larvae in odor + DW (3 cm) region]

### 2.3. Naïve Olfactory Test

Fifty to one hundred larvae were transferred into the midline of test plates. Two and a half microliters of odorant were added on one side and none on the other side. We then counted the number of larvae in the two semicircular areas after 5 min. In other word, a Naïve olfactory test is virtually the same as learning assays, but there is no training before the test. R.I. is calculated with the equation given above.

### 2.4. Naïve Gustatory Test

In this test, a petri dish with a median separator (Thermo Fisher Scientific Inc., Waltham, MA, USA) was used. The control half was filled with 1% agar made with distilled water and the test half with 1% agar made up with 1 M sucrose (SUC) solution, or a 0.1% quinine hemisulfate (QH) solution as described in Honjo’s work [24]. After solidification, 20 larvae were put on each side near the midline and allowed to move for 5 min. Gustatory R.I. is calculated using the larvae numbers on two sides.

### 2.5. Locomotion Assay

Individual larvae were placed on the surface of a plate of 2.5% agar mixed with 1 mL India ink, which showed a contrast black background compared with the larvae’s white color. They were allowed to acclimate for 1 min and video was recorded for 30 sec at 10 frames per sec by using a Moticam3 digital camera (Motic) and Motic Images Plus 2.0 software. Next the video was analyzed using ImageJ and an MTrack2 plug-in, as Varga previously described [25]. The recorded path length was quantified, and locomotion speed was calculated as the distance traveled per min.

## 3. Results

### 3.1. Aversive Olfactory Learning is Impaired by Down-Regulation of Drosophila DD2R in Dopaminergic Neurons

We used the Gal4-UAS binary system [26] to drive the specific expression of DD2R-RNAi transgene in dopaminergic (DA) neurons. DA-specific driver TH-Gal4 [27] was used to cross with UAS-DD2R-RNAi. This RNAi transgene is expected to down-regulate presynaptic DD2Rs (autoreceptors) expressed in DA neurons. Effects of the UAS-DD2R-RNAi transgene have been reported. Draper *et al.* [28] confirmed the specific effects of DD2R-RNAi by quantifying mRNA levels. DD2R-RNAi reduced DD2R transcript, but D1 receptors, including dDA1, did not. In addition, Wiemerslage *et al.* [21] showed that expression of DD2R-RNAi in DA neurons prevents DD2R-mediated protection of DA neurons from toxin-mediated neuron degeneration.

In order to test aversive learning, *Drosophila* larvae were trained to associate quinine hemisulfate (QH; unconditional stimulus, US) with an odor pentyl acetate (PA; conditional stimulus, CS). After 30 min training, the larvae were tested in the presence of odor without QH, as described in the Materials and Methods section and Figure 1. Next, we calculated the Response Index (R.I.), which indicates preference between the odor and odor-free sides [24]. In this assay, an R.I. value larger than the control indicates that larvae are attracted by the odor after training, while an R.I. value smaller than the control means repulsion.

**Figure 2:**
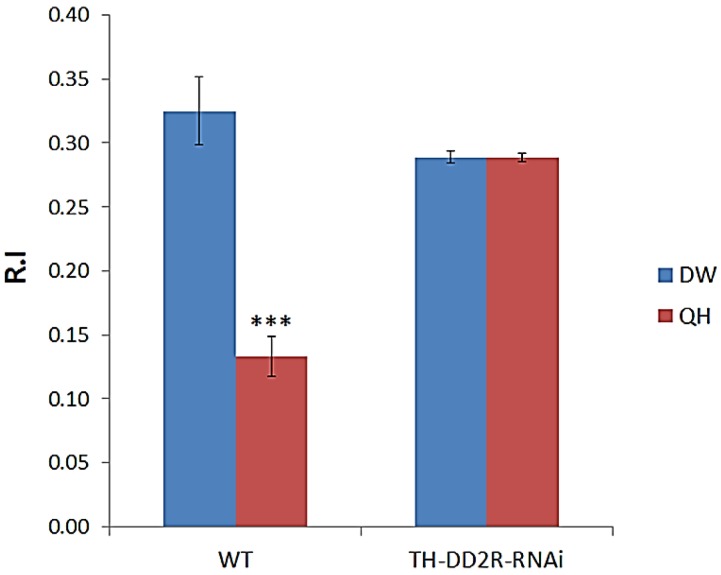
Expression of DD2R-RNAi in dopaminergic neuron-impaired aversive olfactory learning in *Drosophila* larvae. A dopaminergic (DA)-specific driver, TH-Gal4, was used to drive expression of DD2R-RNAi in DA neurons (TH-DD2R-RNAi). Number (n) of separate experiments: wild type (WT) with water (DW) or QH (3), TH-Gal4 × UAS-DD2R-RNAi (TH-DD2R-RNAi) with DW or with QH (10). Student t-test, *** *p* < 0.001.

First, wild type (WT) larvae were trained with PA in the presence of DW, not QH. The R.I value for the DW control larvae was 0.33 ± 0.01 (Figure 2). However, when WT larvae were trained with PA in the presence of QH, the R.I. value was significantly decreased to 0.13 ± 0.01, demonstrating that WT larvae can associate PA (CS) with QH as an aversive stimulus (US). The same learning assay was done using larvae expressing DD2R-RNAi in DA neurons. The R.I. value with QH was 0.29 ± 0.03, which was very similar to that with DW (0.29 ± 0.02), showing a complete impairment of aversive learning. Our data clearly showed that presynaptic DD2Rs are involved in regulation of aversive learning in *Drosophila* larvae.

### 3.2. Appetitive Olfactory Learning is Impaired by Down-Regulation of Drosophila DD2R in Dopaminergic Neurons

**Figure 3:**
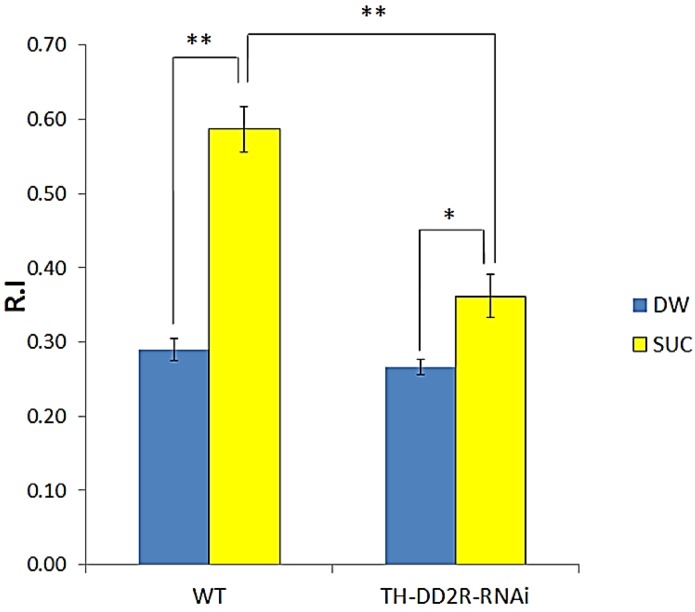
Expression of DD2R-RNAi in dopaminergic neuron-impaired appetitive olfactory learning in *Drosophila* larvae. Number (n) of separate experiments: wild type (WT) with water (DW) or SUC (3), TH-Gal4 × UAS-DD2R-RNAi (TH-DD2R-RNAi) with DW or with SUC (8). Student t-test, * *p* < 0.05, ** *p* < 0.01.

Appetitive learning assays were performed for WT and TH-DD2R-RNAi larvae. This assay was virtually the same as the aversive learning assay apart from using sucrose (SUC) as an appetitive stimulus (US). Wild type (WT) larvae were trained with PA in the absence of SUC (*i.e.*, the DW control). The R.I value for the DW control larvae was 0.29 ± 0.01, while larvae trained with SUC showed an R.I value of 0.59 ± 0.03 (Figure 3). This R.I. value is significantly higher than the DW control, demonstrating that *Drosophila* larvae can learn to associate PA (CS) with an appetitive stimulus SUM (US). The R.I. value for TH- DD2R-RNAi larvae increased (0.36 ± 0.03), compared to that of the DW (0.27 ± 0.02). The results showed that TH-DD2R-RNAi larvae can learn but their appetitive learning was significantly impaired: the R.I. value of 0.36 ± 0.03 is significantly lower than that for WT (0.59 ± 0.03). This means that down-regulation of presynaptic DD2Rs causes partial impairment of appetitive learning. Taken together, our data demonstrate the essential role of presynaptic DD2R autoreceptors in both aversive and appetitive olfactory learning in *Drosophila* larvae.

### 3.3. Aversive Olfactory Learning is Impaired by Down-Regulation of Drosophila DD2R in Mushroom Body (MB) Neurons

**Figure 4:**
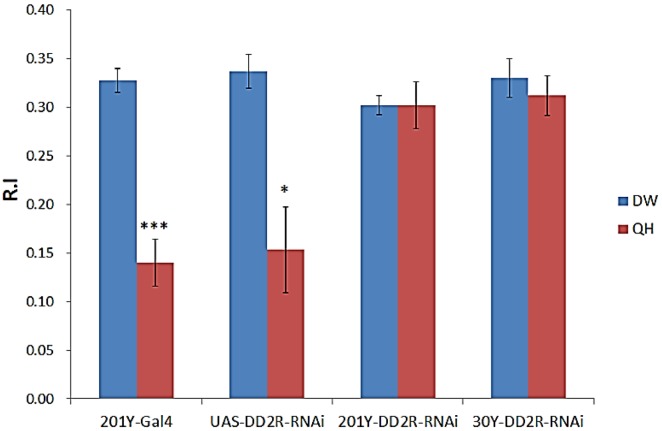
Expression of DD2R-RNAi in the mushroom body-impaired aversive olfactory learning in *Drosophila* larvae. DD2R-RNAi was expressed by using two mushroom body drivers, 201Y-Gal4 and 30Y-Gal4. The 201-Gal4 and UAS-DD2R-RNAi lines were used as controls for this experiment. Number (n) of separate experiments: 201-Gal4 with water (DW) or QH (4), UAS-DD2R-RNAi with DW or QH (3), 201Y-Gal4 × UAS-DD2R-RNAi (201Y-DD2R-RNAi) with DW or with QH (5), and 30Y-Gal4 × UAS-DD2R-RNAi (30Y-DD2R-RNAi) with DW or with QH (6). Student t-test, * *p*<0.05, *** *p* < 0.001.

Mushroom body (MB) is known to be a primary anatomical substrate mediating olfactory learning in the fruit fly [9]. In addition, multiple studies have shown that DA neurons innervate in the MB [7,8]. Therefore, we wanted to down-regulate DD2R in the MB neurons by DD2R-RNAi. Two MB-specific drivers (*i.e.*, 201Y-Gal4 and 30Y-Gal4) were identified [7,24,29,30]. Our previous unpublished data confirmed the expression pattern in the third-instar larval MB by expressing UAS-mCD8::GFP under the control of 201Y-Gal4 and 30Y-Gal4 drivers. Confocal imaging of third-instar larval brains showed that both the 201Y-Gal4 and 30Y-Gal4 strains expressed GFP specifically in the MB neurons [31]. These two Gal4 strains express GFP in different subsets of larval MB neurons [30] and thus are ideal for exploring the functional consequence of DD2R-RNAi expression in the entire gamut of MB neurons. Control larvae (e.g., 201Y-Gal4 and UAS-DD2R-RNAi) were trained with PA in the presence of DW, and the R.I values were 0.33–0.34 (Figure 4). However, when these control larvae were trained with PA in the presence of QH, the R.I. values decreased significantly to 0.14–0.15, demonstrating that the control larvae can associate PA (CS) with QH as an aversive stimulus (US), very similar to WT (Figure 2). The same learning assay was performed using larvae expressing DD2R-RNAi in MB neurons by crossing UAS-DD2R-RNAi with 201Y-Gal4 or 30Y-Gal4 drivers. The R.I. values with QH were 0.3 ± 0.02 and 0.31 ± 0.02 for 201Y-DD2R-RNAi and 30Y-DD2R-RNAi, respectively, showing a complete impairment of aversive learning. Our data clearly showed that postsynaptic DD2Rs in the MB neurons are involved in regulation of aversive learning in *Drosophila* larvae. Two independent MB-specific drivers (201Y-Gal4, 30Y-Gal4) produced very similar results, showing that this learning impairment is not due to a non-specific, genetic background effect.

### 3.4. Appetitive Olfactory Learning is Impaired by Down-Regulation of Drosophila DD2R in Mushroom Body Neurons

**Figure 5:**
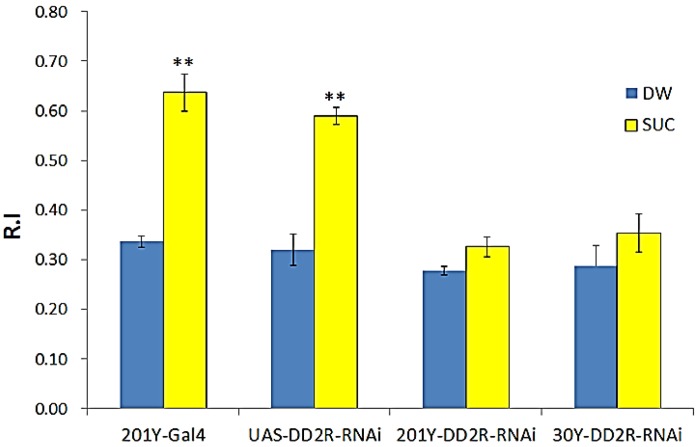
Expression of DD2R-RNAi in the mushroom body-impaired appetitive olfactory learning in *Drosophila* larvae. 201Y-Gal4 and UAS-DD2R-RNAi lines were used as controls for this experiment. Number (n) of separate experiments: 201Y-Gal4 with water (DW) or QH (3), UAS-DD2R-RNAi with DW or QH (3), 201Y-Gal4 × UAS-DD2R-RNAi (201Y-DD2R-RNAi) with DW or with QH (5), and 30Y-Gal4 × UAS-DD2R-RNAi (30Y-DD2R-RNAi) with DW or with QH (6). Student t-test, ** *p* < 0.01.

Appetitive learning assays were performed for 201Y-DD2R-RNAi and 30Y-DD2R-RNAi larvae in addition to control larvae (e.g., 201Y-Gal4, UAS-DD2R-RNAi). 201Y-DD2R-RNAi, and 30Y-DD2R-RNAi larvae were trained with PA in the presence of DW. The R.I values were 0.28–0.29. The larvae trained with SUC showed an R.I value of 0.33–0.35 (Figure 5), which is not significantly different from those with DW, demonstrating that *Drosophila* larvae expressing DD2R-RNAi in MB neurons cannot learn to associate PA (CS) with an appetitive stimulus SUC (US). In contrast, control larvae (Figure 5) R.I. values with SUC were in the range of 0.59–0.64 and thus appetitive learning appears normal in these control larvae. The results showed that down-regulation of postsynaptic DD2Rs in MB neurons caused impairment of appetitive learning. Taken together, our data demonstrated the essential role of postsynaptic DD2Rs in MB in both aversive and appetitive olfactory learning in *Drosophila* larvae.

### 3.5. Sensory and Motor Functions Are Not Affected by Expression of DD2R-RNAi

We showed that both appetitive and aversive olfactory learning behaviors were altered by down-regulation of pre- and postsynaptic DD2R receptors in DA and MB neurons. In general, a potential limitation of our learning assays is that R.I. values can be affected by defects in sensory and motor function due to “ectopic” expression of a transgene—DD2R-RNAi. Therefore, we examined naïve olfactory and gustatory tests in wild type (WT) and crossed lines (TH-DD2R-RNAi, 201Y-DD2R-RNAi, 30Y-DD2R-RNAi), as described previously [24]. Table 1 shows the summary of our results, confirming no differences in sensory function between the fly strains used in this study. To examine motor function, we quantified locomotion speed, as previously described in Varga *et al.* [25]. No difference was observed between fly strains (Table 1). All of the data demonstrate that expression of DD2R-RNAi in DA or MB neurons specifically affects olfactory learning in *Drosophila* larvae, not via alterations in sensory or motor function.

**Table 1:** Sensory and motor responses were tested in the fly lines used in this study. Response Index (R.I.) values of naïve olfactory and gustatory (both aversive and appetitive) tests were obtained as described in the Materials and Methods section. Motor function was tested using locomotion test as described in the Materials and Methods section. No significant difference in olfactory, gustatory, and locomotion speed was observed between wild type and crossed lines. Number (n) of individual experiments repeated.

	WT	TH-DD2R-RNAi	201Y-DD2R-RNAi	30Y-DD2R-RNAi
**Naive Olfactory (R.I.)**	0.34 ± 0.02 (n = 9)	0.31 ± 0.02 (n = 3)	0.36 ± 0.02 (n = 3)	0.35 ± 0.054 (n = 4)
**Naive Gustatory (R.I.) *Appetitive***	0.44 ± 0.10 (n = 3)	0.38 ± 0.05 (n = 3)	0.65 ± 0.13 (n = 3)	0.35 ± 0.08 (n = 3)
**Naive Gustatory (R.I.) *Aversive***	−0.60 ± 0.07 (n = 4)	−0.58 ± 0.05 (n = 3)	−0.43 ± 0.02 (n = 2)	−0.60 ± 0.08 (n = 3)
**Locomotion Speed (mm/min)**	95.56 ± 3.89 (n = 36)	90.53 ± 3.66 (n = 18)	92.78 ± 3.34 (n = 8)	99.74 ± 2.38 (n = 5)

## 4. Discussion

### 4.1. Dopamine D2 Receptor DD2R Mediates Olfactory Learning in Drosophila Larvae

The *Drosophila* D2 receptor DD2R plays an important role in locomotion, aggression, and neuroprotection [21,28,32]. Interestingly, no study has shown whether *Drosophila* DD2R is involved in learning and memory, although dopaminergic (DA) neural circuits and D1 receptors are known to mediate *Drosophila* aversive learning [7,8]. The present study, for the first time, demonstrated that DD2R is involved in olfactory associative learning in *Drosophila* larvae. Further, we showed that both presynaptic and postsynaptic DD2Rs mediate aversive and appetitive learning in the fly larvae as down-regulation of DD2R in DA and mushroom body (MB) neurons resulted in impaired olfactory learning.

### 4.2. Dopamine Signaling in Drosophila Aversive and Appetitive Learning

Multiple studies have proved that dopamine signaling is necessary in *Drosophila* aversive learning [5,6,7]. However, it is uncertain whether dopamine signaling is involved in appetitive learning. Several laboratories reported that DA signaling is not necessary for appetitive learning, which is mediated by another biogenic amine, octopamine [5,7]. In contrast, Selcho *et al.* [8] showed that DA signaling is necessary for appetitive learning as inhibition of DA release resulted in reduced appetitive learning. Furthermore, D1 receptor mutants (e.g., dDA1) showed impaired appetitive learning [8,33].

In the present study, we demonstrated that dopamine mediates not only aversive learning, but also appetitive learning. Both learning behaviors are impaired when DD2R-RNAi is expressed in DA neurons or in MB neurons. Interestingly, aversive learning was completely impaired, while appetitive learning was only partially impaired (Figure 2 and Figure 3) when DD2R-RNAi was expressed in DA neurons. A possible explanation is that the effect of DD2R-RNAi is partial as RNAi down-regulates the target gene expression. Another possibility is that DA is not the only modulatory neurotransmitter mediating appetitive learning; another biogenic amine, octopamine, is involved in appetitive learning [5,7,9]. Therefore, octopamine can mediate appetitive learning to a certain extent even if DA signaling is impaired. In contrast, no modulatory neurotransmitter other than DA is known to be involved in aversive learning.

### 4.3. No Change in Locomotion by Expression of DD2R-RNAi in DA or MB Neurons

Draper *et al.* [28] reported reduced locomotion due to expression of DD2R-RNAi. Our findings do not support their results since the larvae carrying DD2R-RNAi showed no changes in sensory and motor function (Table 1), compared to WT and control fly strains. This discrepancy can be explained through the following reasons. First, it may be related to developmental-specific effects. We used third-instar larvae while adult flies were used by Draper. Second, there are differences in locomotion assays. Draper *et al.* [28] quantified total activity counts, amount of time active, and number of activity-rest bouts. In our study, crawling speed was measured. Third, DD2R-RNAi expression patterns are different. Draper *et al.* [28] used Act5C-Gal or elav-Gal4 to express DD2R-RNAi ubiquitously or pan-neuronally, respectively. In contrast, DD2R-RNAi was only expressed in DA or MB neurons in our study. Therefore, neural circuits affected by DD2R-RNAi can be different, resulting in different behaviors. It is also possible that expression level of DD2R-RNAi is different due to different drivers (e.g., TH-Gal4 *vs.* elav-Gal4).

### 4.4. Role of Presynaptic DD2R Autoreceptors in Olfactory Learning

**Figure 6:**
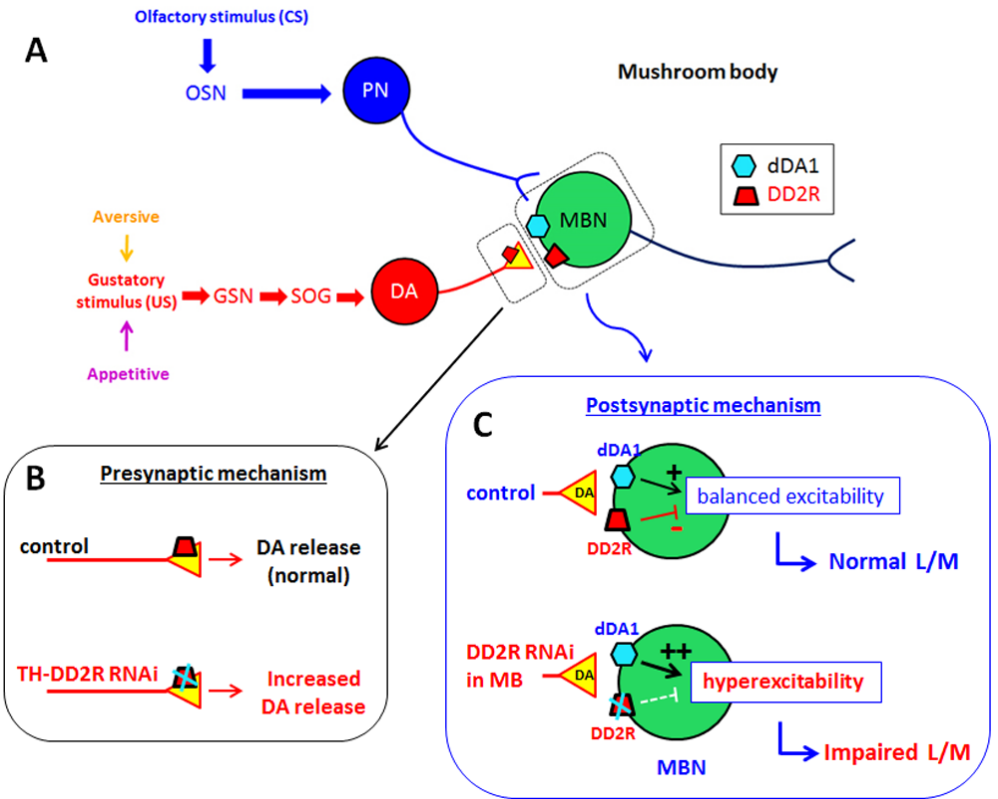
Proposed neuronal and synaptic mechanisms underlying the role of *Drosophila* D2 receptor DD2R in larval olfactory associative learning. **(A)** The diagram shows neural circuits involved in *Drosophila* larval learning. The circuits include three components: (1) Olfactory sensory circuits for CS are comprised of olfactory sensory neurons (OSNs) in antennae, projection neurons (PNs) in antennal lobes, and mushroom body neurons (MBNs). (2) Gustatory sensory circuits for US are comprised of gustatory sensory neurons (GSN), subesophageal ganglion (SOG), dopaminergic (DA) neurons, and MBNs. (3) MBNs serve as coincidence detection of signals from OSNs and GSNs. Thus they can associate olfactory and gustatory signals and mediate olfactory learning in larvae. **(B)** Presynaptic mechanism underlying aversive and appetitive learning. Presynaptic DD2R autoreceptors suppress release of DA at the presynaptic terminals in the MB. Therefore, if presynaptic DD2R function is suppressed, then more DA is released in MB neurons. Increased DA tone in MB neurons impairs both aversive and appetitive learning behaviors. **(C)** Postsynaptic mechanism underlying aversive and appetitive learning. Postsynaptic DD2Rs in MBNs inhibits neuronal excitability as shown in Wiemerslage *et al.* [21]. Therefore, neural circuits associating CS with US maintain balanced excitability. However, DD2R-RNAi in MBNs suppresses postsynaptic DD2Rs and thus neural circuits responsible for learning are over-excited, resulting in impairment of olfactory learning. Our results strongly suggest that DA homeostasis is important for aversive and appetitive learning in *Drosophila* larvae.

Since the identification of *Drosophila* D2 receptor DD2R [19], two studies have revealed the autoreceptor function of DD2R. Vickrey and Venton [20] reported that D2R agonists reduce DA release in the *Drosophila* larval central nervous system. It was also shown that DD2R autoreceptors suppress excitability of DA neurons in *Drosophila* primary neuronal cultures [21]. We showed that DD2R is involved in mediating both appetitive and aversive olfactory learning. DD2R-RNAi in DA neurons down-regulates DD2R autoreceptor function. Thus excitability of DA neurons is increased, leading to an increase of DA release. Our results indicate that excessive DA release impairs olfactory learning. Indeed, Zhang *et al.* [34] showed that olfactory learning is impaired in *Drosophila* DA transporter mutant *fumin*, likely due to increased synaptic DA levels. In contrast, a lack of DA release is known to cause impaired learning in *Drosophila* larvae [8]. Therefore, it appears that homeostatic regulation of DA release by DD2R is important for both appetitive and aversive olfactory learning as either too much or too little synaptic DA causes impaired learning. Taking these facts into consideration, we proposed a model to explain the role of presynaptic DD2R autoreceptors (Figure 6B). Presynaptic DD2R autoreceptors suppress release of DA at the presynaptic terminals in the MB. If presynaptic DD2R function is suppressed, then more DA is released into MB neurons. Increased DA tone in the MB impairs both aversive and appetitive learning behaviors.

### 4.5. Role of Postsynaptic DD2R in Olfactory Learning

We also showed that olfactory learning in *Drosophila* larvae is impaired by down-regulation of postsynaptic DD2R in MB neurons (Figure 4 and Figure 5). As the role of DD2R is inhibitory [2,21], the effects of DD2R-RNAi in MB neurons are expected to increase neuronal excitability, and thus olfactory learning is impaired by hyperexcitability in MB neurons. Our observation might not be consistent with the physiological findings that learning and memory are mediated by enhanced neuronal excitability and synaptic transmission [35,36]. Such well-known examples are long-term facilitation (LTF) and long-term potentiation (LTP) [35,37]. In our study, DD2R-RNAi is expressed throughout the larval stage. Therefore, hyperexcitability is chronic and thus this increased baseline activity interferes with coding new information in the MB. Indeed, Lee and O’Dowd [38] showed that olfactory learning is impaired in *Drosophila* by the chronic increase of excitatory cholinergic synaptic transmission due to the phosphodiesterase gene *dunce* mutation, resulting in increased cAMP levels. Taken together, temporal increases in excitability are key physiological changes underlying associative learning and thus DD2R-RNAi interferes with this change by inducing chronic hyperexcitability in MB neurons.

In addition to DD2R, there are *Drosophila* D1-like receptors (dDA1 and DAMB) that are known to be highly expressed in MB neurons [33]. In fact, dDA1 null mutants showed defects in olfactory learning [8,22]. Since D1-like receptors increase neuronal excitability via the cAMP-PKA signaling pathway [2,9,10], dDA1 mutant MB neurons are less depolarized when DA is released at the synaptic terminal in the MB, and thus cannot mediate olfactory learning. Proper excitability of MB neurons should be maintained by balancing actions of D1- and D2-like receptors in MB neurons.

It has been proposed that the adenylyl cyclase gene *rutabaga* in MB is a coincidence detector for CS and US in *Drosophila* olfactory learning and memory [9,39]. Therefore, on the basis of our results and others’, we propose a model to explain postsynaptic mechanisms underlying aversive and appetitive learning (Figure 6C). Postsynaptic DD2Rs in MBNs inhibit neuronal excitability while dDA1 stimulates neural circuits associating CS with US in MB. DA receptors dDA1 and DD2R regulate AC in MB neurons in the opposite direction to maintain homeostatic balance of MB neuronal excitability, which is an important physiological element for *Drosophila* larval olfactory learning.

## 5. Conclusion

We examined the role of D2-like receptor DD2R in *Drosophila* olfactory associative learning. Our results showed that suppression of presynaptic DD2R autoreceptors impairs both appetitive and aversive learning. Similarly, postsynaptic DD2R in MB neurons appears to be involved in both appetitive and aversive learning.

Our data strongly support the hypothesis that presynaptic DD2R autoreceptors suppress release of DA at the presynaptic terminals in the MB. If presynaptic DD2R function is suppressed, then more DA is released. Increasing DA tone to MB neurons impairs both aversive and appetitive learning behaviors. Postsynaptically, DD2R-RNAi impaired olfactory associative learning most likely by inducing chronic hyperexcitability in MB neurons. Therefore, the role of postsynaptic DD2R is to maintain the proper excitability in MB neurons during learning. Taken together, this study, for the first time, demonstrated that DD2R plays an important role in *Drosophila* olfactory associative learning.
